# Molecular structure of ketoprofen-polyvinylpyrrolidone solid dispersions prepared by different amorphization methods†

**DOI:** 10.1039/d3pm00038a

**Published:** 2024-03-04

**Authors:** Stephen K. Wilke, Chris J. Benmore, Vrishank Menon, Dan Smith, Stephen R. Byrn, Richard Weber

**Affiliations:** a Materials Development, Inc. Evanston 825 Chicago Ave IL 60202 USA swilke@matsdev.com; b X-Ray Science Division, Advanced Photon Source, Argonne National Laboratory Argonne IL 60439 USA; c Improved Pharma West Lafayette IN 47906 USA

## Abstract

Amorphous solid dispersions (ASDs) are a widely studied formulation approach for improving the bioavailability of poorly water-soluble pharmaceuticals. Yet, a complete understanding remains lacking for how specific processing methods may influence ASDs’ molecular structure. We prepare ketoprofen/polyvinylpyrrolidone (KTP/PVP) ASDs, ranging from 0–75 wt% KTP, using five different amorphization techniques: melt quenching, rotary evaporation with vacuum drying, spray drying, and acoustic levitation with either a premixed solution or *in situ* mixing of separate co-sprayed solutions. The co-spray levitation approach enables on-demand compositional changes in a containerless processing environment, while requiring minimal pharmaceutical material (∼1 mg). The structure of all ASDs are then compared using high-energy X-ray total scattering. X-ray pair distribution functions are similar for most ASDs of a given composition (*R*_*x*_ = 0.4–2.5%), which is consistent with them having similar intramolecular structure. More notably, differences in the X-ray structure factors for the various amorphization routes indicate differing extents of molecular mixing, a direct indication of their relative stability against crystallization. Melt quenching, spray drying, and levitation of premixed solutions exhibit some degree of molecular mixing, while the co-sprayed levitation samples have molecular arrangements like those of KTP/PVP physical mixtures. These findings illustrate how X-ray total scattering can be used to benchmark amorphous forms prepared by different techniques.

## Abstract 2

Amorphous solid dispersions of ketoprofen-polyvinylpyrrolidone are prepared by five different techniques; X-ray total scattering reveals differences in their intermolecular structures.

## Graphical abstract

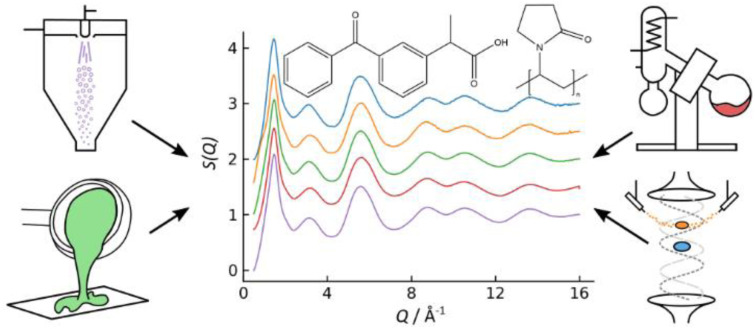

## Introduction

Amorphous formulations are a promising advancement for improving the solubility and bioavailability of poorly soluble active pharmaceutical ingredients (APIs), as compared to their crystalline forms.^1–3^ Amorphous forms are, by definition, thermodynamically unstable with respect to the crystalline state(s), which implies that any method for preparing an amorphous product must bring the material into a state of nonequilibrium. In some of the established amorphization methods – hot melt extrusion,^4,5^ spray drying,^6,7^ cryogenic milling,^8,9^ and compounding with excipients^10,11^ – nonequilibrium states are achieved by thermal quenching, rapid solvent evaporation and supersaturation of a solution, mechanochemical activation, and kinetic barriers to diffusion at a molecular level.^12,13^ One drawback to most of these methods is the amount of thermal or mechanical treatment that must be applied during processing, which can lead to API degradation or other adverse effects.^14^

Recently, acoustic levitation has been demonstrated as an effective amorphization method that avoids elevated temperatures and minimizes the quantity of API necessary for early-stage solubility and toxicity screening.^15–17^ Numerous APIs have been amorphized *via* acoustic levitation, including cinnarizine, carbamazepine, miconazole nitrate, probucol, clotrimazole, ibuprofen, dibucaine, ketoprofen, and clofoctol.^15,18^ Like spray drying, the levitation technique achieves nonequilibrium states *via* solvent evaporation and supersaturation of a solution containing the API. However, because the solution droplet remains stationary during levitation, the technique provides greater versatility for studying the amorphization process parameters. For example, acoustic levitation has revealed how droplet temperature, evaporation rate, and final product morphology correlate with ambient temperature and relative humidity.^19–22^

In crystalline API forms, the physicochemical properties correlate with the molecular structure defined by the crystal lattice. Different crystalline polymorphs can exhibit vastly different properties, with paramount implications for therapeutic applications, which is why polymorph screening is required in regulatory frameworks like ICH Q6A.^23^ Similarly, the properties of an amorphous form also arise from the molecular structure, though, instead of long-range crystalline order, the structure is defined by the network and connectivity between API molecules and, if present, any excipients. For example, amorphous molecular structures that contain greater extents of hydrogen bonding have been found to be more stable against crystallization, as in the case of nifedipine-polymer amorphous solid dispersions (ASDs).^24^ Because there are many methods for preparing amorphous formulations, it is crucial to compare differently prepared products to ensure their similarity.

A few studies have reported differences in thermal stability and/or structure between amorphous formulations prepared by different methods, particularly when comparing heat- and milling-based amorphization. Feng *et al.*^25^ compared the thermal stability and recrystallization behavior of griseofulvin after varying periods of cryogenic milling. Longer milling durations led to increasingly defective crystals, but even after the longest milling time the griseofulvin was distinct from a truly amorphous analogue prepared by melt quenching: the milled product did not exhibit a glass transition (no *T*_g_) and recrystallized at lower temperatures than the melt-quenched form's *T*_g_. In investigations of amorphous indomethacin, both Savolainen *et al.*^26^ and Karmwar *et al.*^27^ observed lower thermal stability against recrystallization for samples made by milling as compared with melt quenching or spray drying. In principal component analyses of their X-ray diffraction and Raman spectroscopy measurements, replicate samples clustered according to their preparation route, suggesting that structural differences arose from the various amorphization methods. Dedroog *et al.*^9^ also observed stability differences for naproxen-polymer ASDs prepared by hot melt extrusion, cryogenic milling, or spray drying. By comparing ASDs containing different polymers, they found that the various amorphization methods each had a different polymer that optimized ASD stability. For the acoustic levitation technique, only one comparison has been reported, finding that 3 : 1 PVP/atazanavir sulfate ASDs prepared by either levitation or spray drying were structurally similar.^17^

In all these prior studies, if milling techniques are excluded from the comparison, the differences reported for products of various amorphization routes are less significant. It is reasonable to expect that different thermal processes for amorphization should yield products with similar molecular structures. From an energy landscape perspective,^28,29^ amorphous structures exist in shallow energy wells, contrasted with crystalline polymorphs that exist in more sharply defined, deep energy wells.

In this study, we expand on this prior literature by comparing acoustic levitation alongside three other amorphization techniques, while extending the investigation to a range of compositions along an ASD binary. We prepare ASDs of ketoprofen (KTP) and polyvinylpyrrolidone (PVP), a well-studied model system,^7,11,30,31^ first with three established techniques: melt quenching, rotary evaporation with vacuum drying, and spray drying. The other two techniques use acoustic levitation with different solution injection methods. We then compare the molecular structure of KTP/PVP ASDs containing 0, 25, 50, or 75 wt% KTP using high-energy synchrotron X-ray total scattering and pair distribution function (PDF) analysis.^18,32^ Our research goal is to benchmark the newer levitation techniques against the more conventional amorphization methods, and X-ray total scattering provides a powerful tool for comparing the intra- and inter-molecular structures of ASDs prepared by different routes and with different drug loadings.

## Experimental

### Sample preparation

**Fig. 1:**
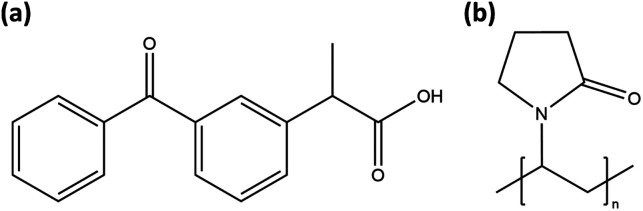
Molecular structures for (a) ketoprofen (KTP) and (b) polyvinylpyrrolidone (PVP).

Samples were prepared from ketoprofen (“KTP,” Millipore Sigma, >98% TLC) and PVP (Kollidon 17 PF, *M*_w_ ∼ 9000, BASF^33^). Molecular structures are shown in Fig. 1. The PVP was not dried prior to use and, based on mass loss if vacuum dried, contained 9.4 wt% absorbed water.

For the first amorphization method, a premixed solution of KTP/PVP was placed in an acoustic levitation instrument (“LevPM”). Solutions were prepared with 0 : 5, 1.67 : 5, 5 : 5, or 10 : 3.33 mg mL^−1^ of KTP : PVP in acetone (0, 25, 50, 75 wt% KTP *vs.* KTP + PVP) by gentle stirring without heating until fully dissolved. A single droplet *ca.* 14 μL (∼3 mm diameter spheroid) was then pipetted into the central acoustic node of a single-axis acoustic levitator. Details of the levitator construction and operation have been reported previously.^34^ The sample levitated for 15–30 min while the solvent evaporated.^17^ The droplet size was recorded using a video camera, and after drying was complete the solid product was retrieved on a piece of polyimide tape.

**Fig. 2:**
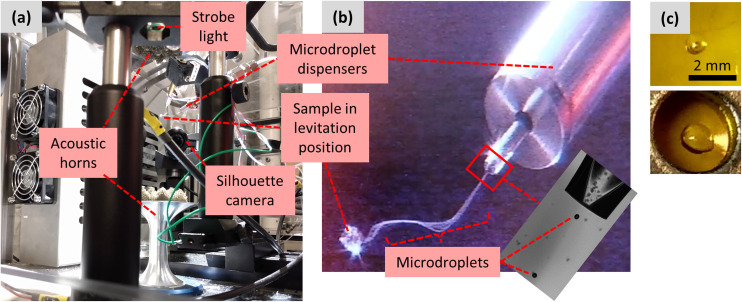
Preparation of samples made by co-spraying KTP and PVP solutions with microdroplet dispensers in the acoustic levitator. (a) Overview of setup with labeled components as described in main text. (b) Picture of one dispenser spraying a continuous series of microdroplets into the main sample held in the levitation position; inset shows a silhouette strobe image of the dispenser tip (40 μm diameter orifice) and two microdroplets traveling toward the sample. (c) Two amorphous solid samples recovered from the levitator after solvent evaporation, each laying on polyimide tape.

The second amorphization method again used the acoustic levitator, but the sample was injected to the central acoustic node of the levitator by co-spraying (“LevCS”) two piezoelectric microdroplet dispensers (MicroFab MJ-AB-01-40). The setup of the dispensers and acoustic levitator is shown in Fig. 2. The acoustic standing waves were produced by a pair of opposing aluminum horns, vertically oriented and driven by Langevin piezoelectric transducers.^35^ The two microdroplet dispensers were positioned ∼60 mm away from the central acoustic axis, pointing at a 45° downward angle toward the central acoustic node of the levitator. The two microdroplet dispensers were connected to separate solutions of 5 mg mL^−1^ PVP in acetone and 10 mg mL^−1^ KTP in acetone, each prepared by gentle stirring without heating. During operation, microdroplets were ejected from the dispenser's orifice.^36^ They appeared visually as a continuous stream that travels toward the acoustic node, where they were captured, and the main sample began to grow from the accumulation of captured microdroplets (Fig. 2b). The quality and consistency of microdroplet dispensing were monitored using a stroboscopic backlight imaging technique.^37^ A strobe light and camera were positioned on opposing sides of the dispenser orifice (Fig. 2a), and the strobe was synchronized to the dispensing frequency to enable observation of the microdroplets as they exited the dispenser's orifice (Fig. 2b, inset). Further details on operating the microdroplet dispensers can be found in the ESI.† For each levitated sample, the microdroplet dispensers operated for 20 min, and the accumulated droplet was held in the levitator for an additional 30–60 min for solvent evaporation.

The third amorphization method used melt quenching (“MQ”). Mixtures of KTP and PVP were heated to 120 °C in scintillation vials suspended in an oil bath. While being heated, the samples were occasionally stirred with a spatula. Upon reaching temperature, heating was continued for 10–15 min and the samples were then cooled to ambient. The 75 wt% KTP sample fully melted at 120 °C and remained fluid during cooling, the 50 wt% KTP mixture formed a highly viscous liquid upon cooling, and the 25 wt% KTP mixture remained solid throughout the process.

The fourth amorphization method used rotary evaporation (rotovap, “RV”) followed by vacuum drying. Solutions were prepared following the same procedure and compositions as for the LevPM samples. Each solution was rotovapped at 30 °C until its viscosity neared the point of being too high to pour out of the flask. At that point, the viscous supersaturated solution was poured onto polyimide tape and transferred to a vacuum chamber for final drying. Attempts were made to instead fully evaporate the solvent in the rotovap, but the resulting product was too viscous to pour out, and scraping caused the amorphous material to crystallize. For this reason, the two-step process of rotovapping and then vacuum drying was implemented.

The fifth amorphization method used spray drying (“SD”). KTP and PVP were added to CH_2_Cl_2_/MeOH (1 : 1) and then stirred until a homogeneous solution formed. The solutions were spray dried using a Buchi B290 spray dryer (inlet temp: 85 °C, aspirator: 100%, pump: 25%).

### X-ray total scattering

High-energy X-ray scattering measurements were performed at Sector 6-ID-D of the Advanced Photon Source (Argonne National Laboratory, Lemont, IL). MQ and SD samples were loaded into 1.9 mm diameter polyimide tubes, and all other samples were placed on thin polyimide tape. A beam of 100.233 keV X-rays was collimated to a 0.5 × 0.5 mm cross section at the sample position, and the diffracted intensity was measured in transmission geometry with a two-dimensional area detector (Varex 4343CT). A sample-to-detector distance of ∼329 mm enabled detection over a range of scattering vector magnitude, *Q*, from 0.5 to 22 Å^−1^. Each X-ray measurement was collected for a duration of 5 min.

The scattering data were reduced to obtain the X-ray total structure factors, *S*(*Q*), following procedures described previously.^38^ Azimuthal integration of the area detector signal was performed in Fit2D,^39^ and structure factors were calculated in GudrunX^40^ using free-atom X-ray atomic form factors.^41^ Background scattering from air and the polyimide sample holders was subtracted, and corrections were applied for X-ray polarization, detector flat field, attenuation, and oblique incidence, sample attenuation, and – though likely insignificant – multiple scattering and X-ray fluorescence.^42^ In GudrunX, the “top hat” convolution^43^ was implemented with a reciprocal space width of 6.0 Å^−1^ and a real space minimum radius of 0.4 Å. Because the levitated samples were quite small, their scattering intensity was weaker in comparison to the MQ and SD samples, so all structure factors were renormalized to the MQ samples. For this renormalizing, a scale factor was applied to each *S*(*Q*) that minimized the root-mean-square difference between it and the corresponding MQ sample over the range 4.2 < *Q* < 16 Å^−1^. This range of *Q* values represents the intramolecular structure of the material^32^ and thus is expected to be similar for samples of the same composition. The resulting scale factors ranged from 0.92 to 1.20.

The differential pair distribution function (PDF), *D*(*r*), was then calculated from each structure factor according to:1
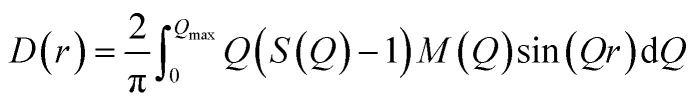
where *M*(*Q*) is a Lorch modification function,^44^ and a *Q*_max_ of 16 Å^−1^ was selected based on difficulty obtaining a flat baseline and reduced signal-to-noise for small samples at larger *Q*. Structural similarity between samples was assessed *via* the differences between their structure factors and PDFs. The PDF difference between each sample and the compositionally similar MQ sample was quantified using the *R*_*x*_ factor:^45^2
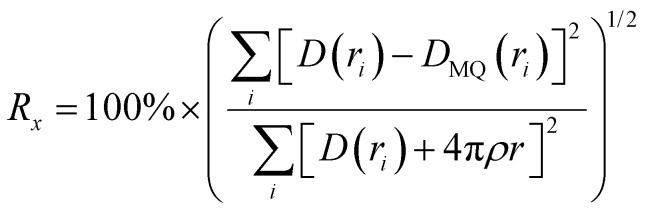


## Results

### Sample morphology

After solvent evaporation in the acoustic levitator, the solid samples were transparent spheroids (Fig. 2c). During solvent evaporation of polymeric solutions in an acoustic levitator,^19^ the initially fluid sample at some point forms a surface skin as the surface layer becomes increasingly viscous (the “lockpoint”). Further diffusion of solvent through the skin and its evaporation leads to a radial pressure gradient that causes surface collapse, resulting in a flattened or nonspherical sample with a porous interior. The visual appearances of the LevPM and LevCM samples here were consistent with this mechanistic process.

### Comparison of amorphization methods

**Fig. 3:**
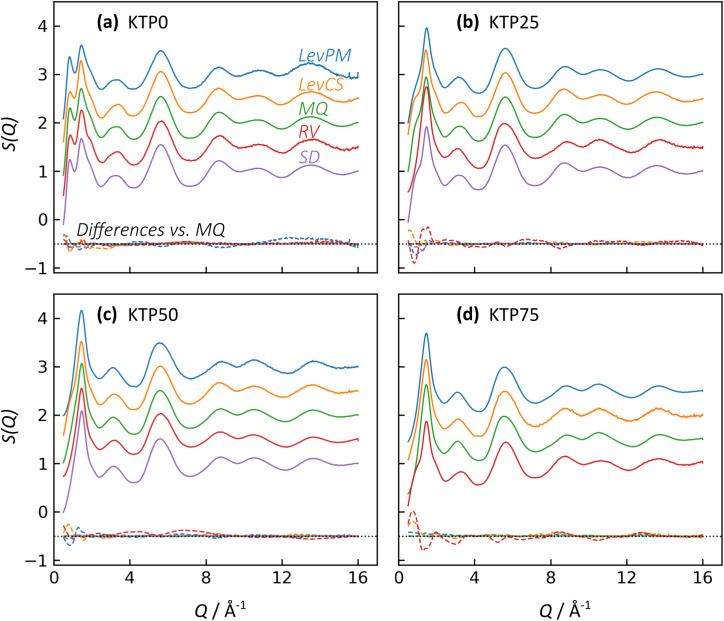
X-ray total structure factors for KTP/PVP amorphous solid dispersions (ASDs) made by five different amorphization techniques, as labeled in (a). (a) 0 wt%, (b) 25 wt%, (c) 50 wt%, and (d) 75 wt% KTP. Structure factors are vertically offset for clarity. The differences between each structure factor and the corresponding melt quench (MQ) sample are shown with dashed curves near the bottom of each plot.

X-ray structure factors, *S*(*Q*), for all KTP/PVP binaries are shown in Fig. 3. The absence of Bragg diffraction peaks indicates that fully amorphous samples were successfully prepared by all five methods: premix levitation (LevPM), co-spray levitation (LevCS), melt quenching (MQ), rotary evaporation (RV), and spray drying (SD). The entire compositional series of 0, 25, 50, and 75 wt% ketoprofen (KTP0, KTP25, KTP50, KTP75) was accessible by all methods except for SD KTP75, which formed a gum and could not be processed.

X-ray total scattering is a useful characterization tool because it can provide structural information on both the intra- and inter-molecular arrangements in amorphous materials.^18,32,46,47^ In *S*(*Q*), the low-*Q* range contains information on the intermediate range order (in real space) that characterizes the amorphous network, while the high-*Q* range represents the local bonding between atoms. For the purposes of comparison here, we select the local minimum in *S*(*Q*) near *Q* = 4.23 Å^−1^ to demarcate these two ranges. For amorphous molecular materials, it is helpful to interpret *S*(*Q*) as a summation of the intra- and inter-molecular interactions. The intramolecular component arises from bonds within both KTP and PVP, and the intermolecular component represents how pairs of molecules – KTP/KTP, KTP/PVP, or PVP/PVP – are arranged. This way of interpreting *S*(*Q*) is useful in making comparisons between samples. For example, ASDs of the same composition made by different processing methods should have identical intramolecular contributions to *S*(*Q*), but we aim to assess whether their intermolecular packing (network structure) is different. The high-*Q* range is mostly influenced by intramolecular bonding and thus, for this example, would be similar, while the network differences would manifest as such in the low-*Q* range.^32^

In Fig. 3, the first two diffraction peaks are near *Q*_1_ = 0.85 and *Q*_2_ = 1.46 Å^−1^, and these peaks represent the intermolecular network.^18^ The first peak is most notable in KTP0 and decreases in intensity as KTP content increases, so *Q*_1_ is associated with the PVP packing arrangement. In the PVP molecule, the distance between the –C–C– backbone and the outer C atoms of each lactam group is ∼3.9 Å. Assuming that the lactam groups are randomly oriented in directions orthogonal to the molecule backbone, the spacing between adjacent PVP backbones would be roughly 3.9 × 2 = 7.8 Å, which corresponds approximately with 2π/*Q*_1_ = 7.4 Å. The second peak's intensity increases with KTP content, so this increase is associated with the KTP molecular packing.

The structure factors for same-composition ASDs made *via* different amorphization methods are similar except for small differences at low-*Q*. Since MQ is an established amorphization technique and is inherently solvent-free, it is used here as a reference for comparing the other methods. The differences between each structure factor and the corresponding MQ sample are shown near the bottom of each plot in Fig. 3. In general, the magnitudes of the difference functions are small and non-systematic (in contrast to compositional differences, discussed later).

For the *Q* > 4.23 Å^−1^ range, the samples with the largest differences (*vs.* MQ) are the RV ASDs (all compositions) and LevPM KTP0. For RV, the differences likely arise from residual acetone solvent that was not fully evaporated, which would result in differences in the measured intramolecular structure since molecules in addition to KTP and PVP are present. During preliminary RV trials, complete solvent evaporation in the rotovap was possible, but the resulting product was too difficult to remove from the rotovap flask, and scraping to remove the product caused crystallization. To avoid this problem, rotovapping was stopped before full solvent evaporation while the sample was still sufficiently fluid to pour onto polyimide tape. The sample was then dried under partial vacuum at 25 °C, but perhaps not long enough to extract all solvent. In the case of LevPM KTP0, the ASD sample recovered from the levitator was exceptionally small (∼0.5 mm diameter) compared to the others (>1 mm diameter), and the X-ray data reduction steps are more difficult for smaller samples, which can lead to a non-flat baseline in *S*(*Q*).

In comparing the *Q* < 4.23 Å^−1^ range, SD is the most similar to the MQ reference, while the LevPM and LevCS show some slight variation. These variations suggest the possibility of some differences in molecular packing, which are discussed later. This low-*Q* range can be affected by imperfect X-ray data corrections for the small samples obtained from levitation, so future work could use measurements on multiple replicate beads obtained from the levitator.

**Fig. 4:**
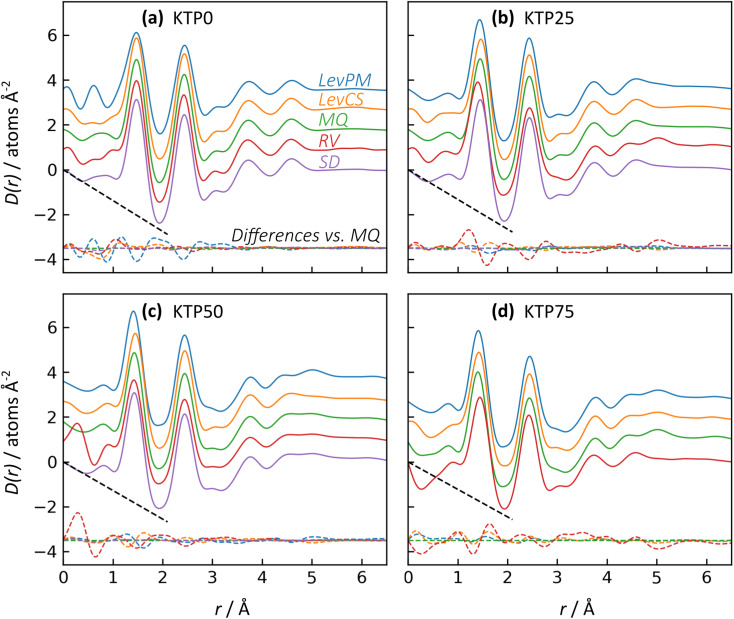
X-ray differential pair distribution functions (PDFs) for KTP/PVP ASDs made by five different amorphization techniques, as labeled in (a). (a) 0 wt%, (b) 25 wt%, (c) 50 wt%, and (d) 75 wt% KTP. PDFs are calculated with *Q*_max_ = 16 Å^−1^ and are vertically offset for clarity. The dashed black lines indicate the anticipated low-*r* slope of −4π*ρ*, where *ρ* is the atomic number density. The differences between each PDF and the corresponding MQ sample are shown with dashed curves near the bottom of each plot.

The X-ray pair distribution functions (PDFs) for all ASDs are shown in Fig. 4. The first few peaks in the PDF can be matched qualitatively to the anticipated bond lengths for KTP and PVP molecules: *r* ∼ 1.44 Å for C–(C, O, N) bonds, and *r* ∼ 2.44 Å for next-nearest neighbor C–C distances, as well as the diameter of KTP's two benzene rings and the PVP monomer's 5-membered lactam. The H–(C, O, N) bonds are expected near *r* = 1 Å but are conflated with oscillations in the low-*r* region arising from imperfect X-ray data corrections in the structure factors, including deviations from the free atom X-ray form factor approximation.^48^ Note that these assignments are not exact, as intermolecular correlations are also contributing to the PDF. The PDFs agree well with the expected low-*r* slope given by −4π*ρ*, where *ρ* is the atomic number density (Fig. 4, dashed black lines). For KTP and PVP, *ρ* = 0.0938 and 0.1097 atoms Å^−3^, based on a density of 1.2 g cm^−3^ for each. The densities of the ASDs were calculated using a linear interpolation of the pure endmembers.

Like with the structure factors, the PDF for each ASD is compared against the corresponding MQ sample as a reference, and the differences are shown in dashed lines near the bottom of the plots in Fig. 4. The *R*_*x*_ factors (eqn (2)), listed in Table 1, are used to quantify the differences for these pairwise comparisons, with smaller *R*_*x*_ indicating greater similarity. The *R*_*x*_ values are calculated for the range 1 < *r* < 6 Å to avoid *r* < 1 Å, which is influenced by the challenges in data reduction for small samples.

**Table 1:** *R*_*x*_ factor (in %) for the X-ray PDF difference between each sample and the corresponding MQ sample (Fig. 4). The red shading intensity represents relative magnitude

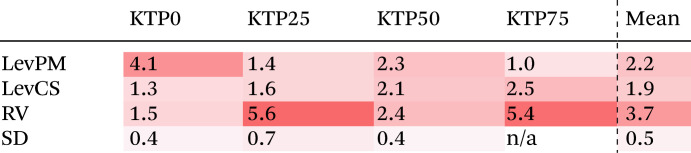

The PDF differences for a given composition are small (*R*_*x*_ = 0.4–2.5%), except for LevPM KTP0 and RV KTP25 and KTP75 (*R*_*x*_ = 4.1, 5.6, and 5.4%, respectively). These exceptions are the same as those already discussed in the context of X-ray structure factors. In general, the SD samples are most structurally similar to MQ (mean *R*_*x*_ = 0.5%), followed by the LevCS and LevPM (mean *R*_*x*_ = 1.9 and 2.2%). RV samples are the most different (mean *R*_*x*_ = 3.7%). For example, the first peak position varies less than ±0.01 Å among all samples except RV, which differs by up to 0.5 Å compared to the others (*e.g.*, KTP25 in Fig. 4b).

### Comparison of ASD compositions

**Fig. 5:**
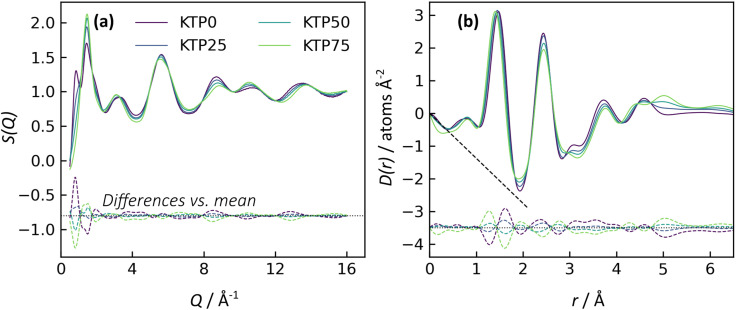
(a) X-ray total structure factors and (b) PDFs for KTP/PVP ASDs made by melt quenching. PDFs are calculated with *Q*_max_ = 16 Å^−1^, and the dashed black line indicates the anticipated low-*r* slope of −4π*ρ*, where *ρ* is 0.10965 atoms Å^−3^ corresponding to the 0 wt% KTP composition. The differences between each curve and the mean of the four compositions are shown with dashed curves near the bottom of each plot.

To examine the structural differences due to compositional changes, the X-ray structure factors and PDFs for the MQ ASDs are shown in Fig. 5. In both *S*(*Q*) and the PDF, the curves for the four different compositions cross at common points. These isosbestic points^49,50^ are indicative of a series of measurements with changing populations of two species or two contributions, and the appearance of isosbestic points here is similar to other studies on structural changes across a binary compositional series.^51^ In the PDFs, the trend in density is difficult to discern from the low-*r* region but can be inferred from the minimum in *D*(*r*) near *r* = 1.92 Å. The value of *D*(*r*) at this minimum increases with KTP content, suggesting a decrease in density consistent with the calculated densities for pure KTP and PVP.

Near the bottom of the plots in Fig. 5, the differences between each sample and the mean of the four samples are shown. These difference functions show systematic variations, in contrast to the non-systematic variations observed between amorphization methods for a single composition (*cf.*Fig. 3 and 5a, or Fig. 4 and 5b). The differences in the compositional series’ *S*(*Q*) are evident for both the network structure (*Q* < 4.23 Å^−1^, mainly) and intramolecular bonding (*Q* > 4.23 Å^−1^). In the PDFs, the first peak position moves to slightly smaller *r* as KTP content increases. The *R*_*x*_ for PDF compositional differences is ∼2.8% per 25 wt% KTP variation, which is a larger *R*_*x*_ than all PDF comparisons of different amorphization methods except for the three outliers mentioned (LevPM KTP0; RV KTP25 and KTP75).

## Discussion

KTP/PVP was selected for this structural study because it has been widely investigated in the context of amorphous formulations’ properties and behavior. ASDs of KTP/PVP have been shown previously to have improved dissolution rates in comparison to crystalline ketoprofen. Spray dried KTP/PVP ASDs with 20 wt% KTP had dissolved 40% of their available KTP after 5 min, in comparison to only 15% for tests with pure KTP.^7^ Prior work also indicates that dissolution rates depend on the KTP : PVP ratio. ASDs with 1 : 1 KTP : PVP exhibited a dissolution efficiency more than double that of pure KTP, and dissolution rate continued to increase with additional PVP content.^31^

### Acoustic levitation with microdroplet dispensing

Acoustic levitation can be a useful technique for research in amorphous pharmaceutical formulations. Because its use for this application is relatively recent (2011),^15^ we provide a brief discussion of its operation and the sample manipulation capabilities that are explored in this work.

Single-axis acoustic levitation is a containerless processing technique that uses the force exerted by standing sound pressure waves to counteract the force of gravity, thereby yielding stable levitation.^34,52^ By avoiding container-induced heterogeneous nucleation, levitation can create pathways to nonequilibrium states *via* solvent evaporation, solution supersaturation, supercooling, or chemical processes. In this work, solvent evaporation during levitation leads to avoidance of crystal nucleation, resulting in an amorphous product. The effects of temperature and relative humidity on such processes can also be studied by working in an environmental control chamber, and the stationary position of the sample simplifies observation and *in situ* characterization by other experimental probes.

Most often, liquid samples are placed in the levitation position (*i.e.*, one of the acoustic nodes, between regions of high sound pressure) by a manual pipette or a needle connected to a syringe pump. To switch compositions, a new solution must be loaded in the pipette or needle. An alternative approach, demonstrated in this study, is to co-spray from several microdroplet dispensers connected to different solutions. The basic operation of piezoelectric “drop-on-demand” microdroplet dispensers has been described and optimized,^53,54^ but their use with acoustic levitation has not yet been reported in detail.^55^ This technique could enable several additional experimental options. First, amorphous forms can be produced using minimal material: ∼1 mg, compared to ∼10 mg for spray drying.^56^ Second, the flowrate for different dispensers can be changed on-demand to (i) automate sequential preparation of samples across a compositional range, (ii) vary the composition within a single sample, or (iii) perform chemical processes in the containerless environment, such as a pH adjustment.

One challenge to the co-spraying process was that not all microdroplets were entrained to the levitation position. Some fraction of the microdroplets drifted away from the levitation axis or traveled to the acoustic nodes adjacent to the central position. While the severity of this effect was minimal, it introduces some uncertainty to the compositions obtained by co-spraying because the microdroplet capture rate was sometimes different for the two dispensers.

### Molecular mixing in amorphous dispersions

An amorphous form can exhibit different properties depending on whether the API and excipient(s) are merely physically mixed, or bonded at a molecular level. In a physical mixture, large domains of API molecules exist separately from large domains of excipient, and this arrangement can increase the API's tendency to crystallize during storage. In contrast, a molecular mixture is characterized by increased intermolecular bonding between API and excipient, which can stabilize the ASD. For KTP/PVP, Di Martino *et al.*^30^ compared binary forms prepared either by physical mixing, or solvent evaporation from a KTP/PVP solution. The physical mixture did not generate an amorphous form. For solvent evaporation, no crystallinity was observed even after one year of storage. These differences were correlated with NMR measurements showing no significant KTP-PVP interactions in the physical mixture, while the solvent-evaporated form exhibited hydrogen bonding between KTP and PVP molecules, suggesting some degree of mixing at a molecular level.

**Fig. 6:**
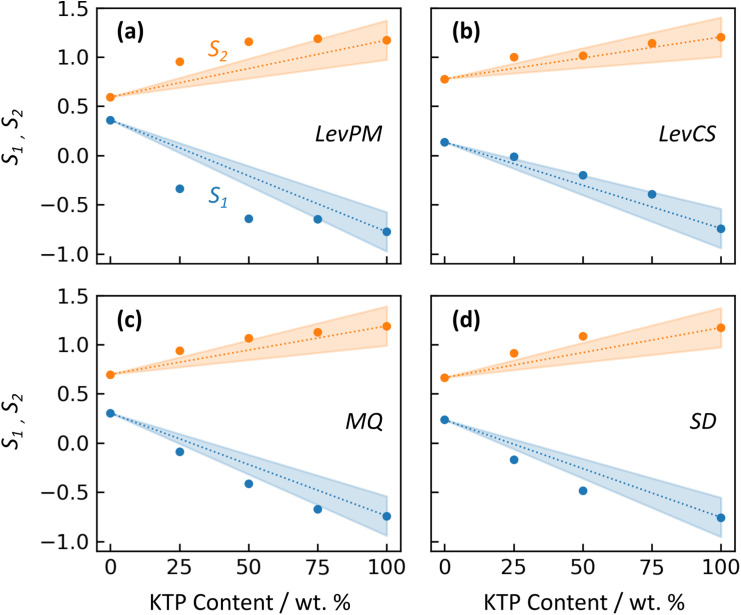
Intensities of the first and second peaks in the X-ray structure factors, *S*_1_ (blue) and *S*_2_ (orange), *versus* the KTP content in ASDs. Data markers are shown for four amorphization methods: (a) LevPM, (b) LevCS, (c) MQ, and (d) SD. Linearity between the pure endmembers is shown by dotted lines, with the shaded wedges representing the uncertainty arising from *S*_1_ and *S*_2_ for KTP100.

In this study, the X-ray structure factors can be used to qualitatively compare the extent of molecular mixing between amorphization methods. Because intermolecular structure is strongly reflected by the first two diffraction peaks, their intensities *S*_1_ and *S*_2_ (near *Q*_1_ = 0.85 and *Q*_2_ = 1.46 Å^−1^) are plotted *vs.* KTP content in Fig. 6 for the different amorphization routes. (RV is not included in this comparison due to its structural – and likely compositional – differences.) For Fig. 6, peak intensities for amorphous ketoprofen (KTP100) are taken from prior X-ray measurements by Benmore and Weber,^15^ who used a LevPM technique. The structure factor of their KTP100 is compared with this study's LevPM in Fig. S1 in the ESI.†

For a physical mixture (PM), the X-ray scattering for a binary of KTP and PVP would be the same as a compositionally-weighted linear combination of the pure endmembers’ scattering,^51,57^ approximately:

3 *S*_PM_(*Q*) = *w*_KTP_*S*_KTP_(*Q*) + (1 − *w*_KTP_)*S*_PVP_(*Q*)

where *w*_KTP_ is the weight fraction of KTP. This relationship would result in *S*_1_ and *S*_2_ each exhibiting a linear trend *vs.* composition, which is shown in Fig. 6 with dotted lines. The slopes of these lines have some uncertainty, however, because KTP100 data is only available for LevPM, which is used in Fig. 6b–d for the other amorphization methods. This uncertainty in *S*_1_ and *S*_2_ is *ca.* ±0.2 (marked by the shaded wedges), based on how much the KTP0 *S*_1_ and *S*_2_ vary between amorphization methods.

The ^13^C NMR findings of Di Martino *et al.*^30^ have shown that the degree of hydrogen bonding between KTP and PVP is highly influenced by the manner in which they come into contact, *i.e.* whether heterogeneous domains of KTP persist within the PVP polymer (with little or no interaction), or there is significant hydrogen bonding at the molecular level. To investigate this further, we can compare the experimental *S*_1_ and *S*_2_ data markers with the linear relationship expected for the hypothetical physical mixtures. For LevPM, MQ, and SD, the peak intensities deviate from the linear relationship with composition, indicating some degree of molecular mixing. This deviation suggests bonding between KTP and PVP, resulting in an amorphous solid in a deeper energy well than that of a physical mixture, making it more stable and less likely to crystallize. LevPM is the most nonlinear (*i.e.*, *S*_1_ and *S*_2_ lie farthest outside the shaded wedges in Fig. 6), followed by SD and then MQ. It is possible that a greater degree of molecular mixing is achieved in LevPM and SD because of the higher molecular mobility in solution, as compared to the more highly viscous melts for MQ. (For MQ samples in this study, heating was limited to 10–15 min with occasional stirring, so molecular mixing would depend on diffusion for those samples.) For LevCS, the peak intensities fall within the uncertainty for a physical mixture's linear trend, suggesting that the LevCS ASDs have minimal or no significant mixing at a molecular level. One possible explanation for this finding is that the co-sprayed microdroplets of KTP and PVP solutions (each *ca.* 0.015 nL in volume) may lose most of their solvent prior to reaching the levitation position, since their large ratio of surface area to volume could cause fast evaporation. In this scenario, separate domains of highly viscous KTP and PVP would be deposited onto the main sample, and molecular mobility may be too low to achieve molecular mixing. If this is the case, the co-spraying technique must be modified to become useful for ASD development. For example, switching to a solvent with lower vapor pressure (*e.g.*, ethanol, instead of acetone as used here) may help by lowering the microdroplets’ viscosity at the point of reaching the levitation position, thereby improving molecular mixing.

### ASD stability and hydrogen bonding

This study has focused on benchmarking the molecular structure of ASDs prepared by different methods. To compare ASD stability, it is helpful to relate the structure measurements to thermal analysis. Differential scanning calorimetry is commonly used to assess glass transition temperatures (*T*_g_), crystallization, and melting of ASDs. No DSC measurements were conducted on the ASDs in this study, so prior studies are used for context and discussion. In Di Martino *et al.*'s report on KTP/PVP binaries,^30^*T*_g_ for ASDs ranged from 178 °C for pure PVP to −3 °C for pure KTP, and the glass transition decreased as the KTP loading increased. Browne *et al.*^7^ found similar results for KTP/PVP, with *T*_g_ decreasing from 166 °C for pure PVP (a different type than used by Di Martino *et al.*) to 94 °C for an ASD with 20% KTP.

Dedroog *et al.*^9^ conducted thermal analysis on ASDs containing 35% naproxen in polyvinylpyrrolidone vinyl acetate (PVP-VA), to explore differences for spray drying, hot melt extrusion, or cryo-milling preparations. These ASDs made by different methods exhibited different *T*_g_'s and widths of the glass transition. Compared to the spray dried samples, hot melt extruded ASDs had a *T*_g_ 5–10 °C higher and a narrower glass transition, which indicates greater homogeneity. The cryo-milled samples exhibited two *T*_g_'s, indicative of ASD phase separation into polymer- and drug-rich domains. Such phase separation has been correlated with stronger tendency toward crystallization, for example when ASDs containing PVP are exposed to humid storage conditions.^58^ In this study, it is likely that the LevCS samples may have a higher tendency toward crystallization than ASDs prepared by the other amorphization routes, since the LevCS X-ray scattering suggests they are more similar to physical mixtures of KTP and PVP (*i.e.*, they contain drug-rich domains, like those found in phase separated ASDs). Thermal analysis of ASDs obtained by levitation will be explored in future work, to better correlate their molecular structures with stability against crystallization.

Understanding the extent of hydrogen bonding in molecular mixtures is useful for designing ASDs with improved stability against API crystallization. For example, ASD stability can be optimized by selecting a polymer that has higher levels of hydrogen bonding with the API.^11^ In studies on ASDs containing either nifedipine or ibuprofen, better thermal stability was observed when API-polymer interactions included greater degrees of hydrogen bonding.^24,59^ For the ASD binaries in this study, hydrogen bonding is expected between KTP carboxyl and PVP carbonyl groups (Fig. 1), based on past work.^30^ However, it is difficult to directly identify hydrogen bonding contributions from the PDFs because of the overlapping intra- and inter-molecular correlations. In future work, KTP/PVP molecular structure models will be refined based on the X-ray scattering, using empirical potential structure refinement^60,61^ (EPSR). In a previous study, we used EPSR to model the hydration of PVP and quantify the hydrogen bonding between water–water and water-PVP molecules.^62^ The concentration of hydrogen bonding was found to increase linearly as water content increased from 2 to 12.3 wt%, with more hydrogen bonds for water–water than for water–PVP pairs. This provides an example of how such models make it possible to calculate specific hydrogen bonding interactions, which complement the X-ray structure and thermal analysis measurements.

## Conclusions

Amorphous solid dispersions of KTP/PVP have been prepared using five different amorphization routes, including established techniques such as melt quenching and spray drying, as well as newer ASD development approaches using acoustic levitation. We demonstrated a new technique that uses drop-on-demand microdroplet dispensers with the acoustic levitator to enable solution mixing or chemical processes in a containerless environment. The molecular structures of these ASDs were then investigated with X-ray total scattering. Pair distribution functions for most samples of a given composition are similar (generally *R*_*x*_ = 0.4–2.5%), reflective of their similar intramolecular structure. The largest structural differences were observed for the rotovap method, which may be due to retained solvent in the samples.

Differences in the low-*Q* range of the X-ray structure factors indicate variability in the extent of molecular mixing for different amorphization routes. Notably, ASDs prepared by co-spraying KTP and PVP solutions into the acoustic levitator are indistinguishable from the expected compositional trend for physical KTP/PVP mixtures. This suggests a possible limitation of the co-spray technique for obtaining molecularly mixed ASDs. However, if this problem arises from too rapid solvent evaporation, then co-spraying may still be useful for solutions with lower vapor pressure solvents.

Future work will focus on molecular modeling of KTP/PVP to quantify the molecular mixing and better understand hydrogen bonding interactions, which have previously been shown to improve ASD stability against crystallization.

## Conflicts of interest

There are no conflicts to declare.

## Supplementary Material

PM-001-D3PM00038A-s001
