# Supplementary material for: Molecular structure of ketoprofen-polyvinylpyrrolidone solid dispersions prepared by different amorphization methods
Source: RSC Pharm. 2024 Mar 4;1(1):121–31. doi: 10.1039/d3pm00038a (PMC11024667; doi:10.1039/d3pm00038a)
Supplement: PM-001-D3PM00038A-s001 [file PM-001-D3PM00038A-s001.pdf]

Electronic Supplementary Material (ESI) for RSC Pharmaceutics.
This journal is © The Royal Society of Chemistry 2024

Supplementary Information for:

Molecular structure of ketoprofen-polyvinylpyrrolidone solid dispersions prepared by different amorphization methods

Stephen K. Wilke, Chris J. Benmore, Vrishank Menon, Dan Smith, Stephen R. Byrn, Richard Weber

Additional operating notes on microdroplet dispensers with the acoustic levitator

Each microdroplet dispenser comprises a thin glass capillary with a 40 μm diameter orifice at the tip, and part of the capillary is enclosed by a ring of piezoelectric material. An electrical waveform is sent to the piezoelectric element, and the transition between low and high voltages causes a mechanical constriction of the capillary, which squeezes out the contained fluid in a series of microdroplets, each approximately 0.015 nL in volume. Each dispenser is connected to a reservoir of solution via plastic tubing with an inline filter, to block small debris from traveling to and clogging the orifice, and the reservoirs are kept under a slight back pressure that is necessary for the microdroplet formation at the capillary orifice.[36]

To achieve a steady state mode of microdroplet dispensing, the waveform applied to the dispenser must be optimized for the solution characteristics. A simple unipolar waveform can be defined by a high voltage, low voltage, rise time, fall time, and the dwell duration at each of the voltages. This waveform is then repeated at a specified frequency, typically 100's to 1000's Hz. For the acetone solutions used in this study, finding a suitable waveform was challenging due to acetone's low viscosity and low surface tension compared to more typical solvents for microdroplet dispensers (e.g., ethanol). After exploring various unipolar and bipolar waveforms, a sinusoidal waveform was found to produce the most stable dispensing. Optimal microdroplet injection to the levitation position was achieved by using a sinusoidal waveform with an amplitude of 17-24 V and a period of 96 μs. Discrete single periods of this waveform were applied to the dispensers at frequencies in the range of 167-1000 Hz, with the specific frequencies chosen so that the co-spraying would achieve the target sample composition.

Several challenges were encountered during the co-spraying levitation process. Successful formation and ejection of microdroplets at the dispenser orifice was highly sensitive to the applied back pressure on the reservoir, and imperfect pressure settings would lead to either air ingestion to the capillary via the orifice, or excess wetting of the orifice by the solution. In both cases, microdroplet formation then became inconsistent until the air bubbles were flushed out and/or excess solution wiped away. A second issue was that when the dispensers were not in use (e.g., between dispensing periods for successive samples), the solution at the orifice would undergo solvent evaporation, and the solute would dry around the orifice into a crust that prevented stable microdroplet formation. For this reason, the orifices required a simple solvent rinse and gentle wiping before each use (without removing them from the setup).

Figures

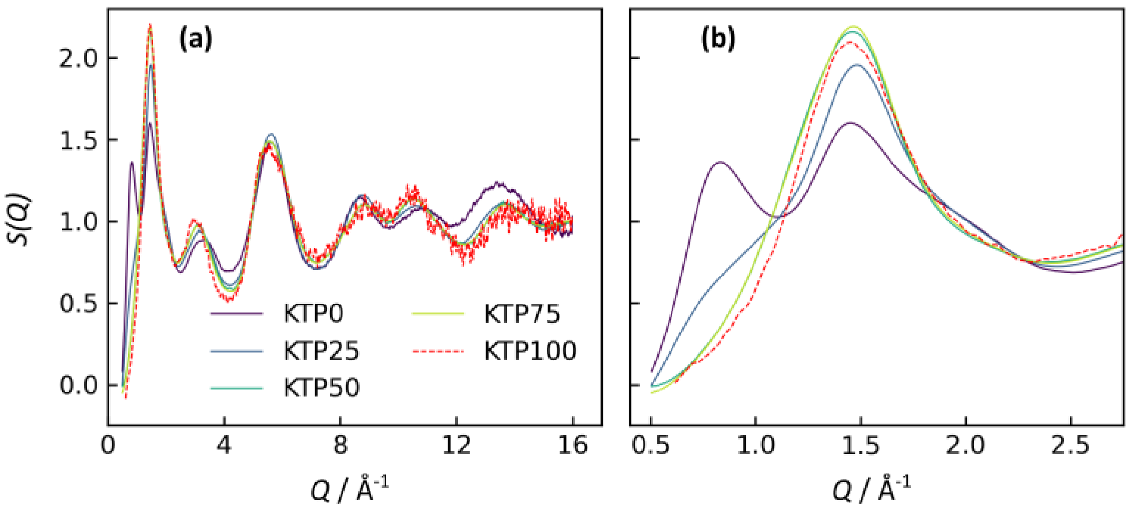

Fig. S1. X-ray total structure factors for ketoprofen/PVP amorphous solid dispersions made by acoustic levitation of premixed solutions (“LevPM”), for 0, 25, 50, and 75 wt. % ketoprofen (KTP). Also shown is amorphous pure ketoprofen (“KTP100”) from Benmore and Weber[15], prepared by the same method but measured with a much lower X-ray beam flux, leading to smaller signal-to-noise in the structure factor. (a) Full Q range, and (b) the first two peaks in the low-Q range that contain structural information on the molecular network and packing. Note: KTP100 has been renormalized slightly (+10%) based on structural modeling that will be detailed in a future report. The magnitude of this renormalization is similar to that used for other LevPM samples, as described in the main text.
